# Perceived Stress Among Nurses at a Tertiary Care Teaching Hospital in Saudi Arabia During the COVID-19 Pandemic

**DOI:** 10.7759/cureus.55433

**Published:** 2024-03-03

**Authors:** Sabeena M Rasheed, Lamees S Bakhsh, Reem S Alhameedi, Sharifah Mohidin

**Affiliations:** 1 Department of Nursing, Faculty of Medicine, King Abdulaziz University, Jeddah, SAU; 2 Department of Nursing, King Fahad Medical City, Riyadh, SAU

**Keywords:** workload, pss score, covid-19, stress, nurses

## Abstract

Background: The stress level of nurses, especially frontline staff, directly impacts their physical and mental health as well as work efficiency. However, few studies have been conducted to understand the stress level of nurses related to the coronavirus disease 2019 (COVID-19) pandemic.

Aim: The aim of this study is to assess the level of perceived stress among nurses at a tertiary care teaching hospital (King Abdulaziz University Hospital, Jeddah) in Saudi Arabia during the COVID-19 pandemic.

Methods: A cross-sectional survey-based descriptive study involved 1044 nurses working at a tertiary care/teaching hospital in Saudi Arabia. The Perceived Stress Scale (PSS) was used to assess nurses' stress levels. The study was conducted in July 2021 by selecting clinical nurses from different nursing units. Considering the current situation of social isolation, an online cross-sectional survey using Google Forms was used. Data analysis was performed using IBM SPSS Statistics for Windows, Version 21 (Released 2012; IBM Corp., Armonk, New York, United States).

Results: Out of 1044 nursing professionals, 93 (8.9%) reported experiencing low levels of stress, 915 (87.6%) reported moderate levels of stress, and 36 (3.4%) reported high perceived stress during the COVID-19 pandemic. The study found that gender (p=0.001) and nationality (p=0.016) had significant effects on stress levels. The three major causes of stress identified by the nurses were workload, lack of resources, and fear of contracting COVID-19.

Conclusion: Job-related stress continues to be an issue in the nursing workforce. The effects of stress experienced by nurses impact job satisfaction and retention, which, in turn, can affect the quality of patient care. This study highlights that nurses in a tertiary care (COVID care) teaching hospital in our setting often experience stress and anxiety during the pandemic. It is worth noting that this is the first study conducted to understand the level of stress perceived by nursing staff during the COVID-19 pandemic in our current study setting. The findings indicate that a considerable number of nurses experienced moderate levels of stress during COVID-19, which can have implications for patient care. Recognizing and understanding the job-related stressors of nurses can help nurse leaders implement more effective strategies aimed at reducing nurses' stress and subsequently improving patient outcomes.

## Introduction

Coronavirus disease 2019 (COVID-19) is a respiratory disease caused by a novel coronavirus (nCoV) that was first identified in Wuhan, China, in December 2019. The outbreak was linked to the South China local Huanan seafood market in Wuhan, Hubei Province, China [1]. Since then, COVID-19 has become a global pandemic, leading to significant challenges for healthcare workers (HCWs) worldwide. The impact on HCWs includes increased demand for medical and nursing staff, rising costs for personal protective equipment (PPE), diagnostic tests for HCWs, beds, and ventilators, as well as a higher mortality rate [2].

The World Health Organization (WHO) declared COVID-19 a Public Health Emergency of International Concern on January 30, 2020, as the disease rapidly spread and reached a pandemic level. As of February 11, 2022, the WHO reported a staggering number of confirmed cases and deaths due to COVID-19 globally [3]. The psychological toll on HCWs during the COVID-19 pandemic is evident from various studies. A meta-analysis conducted by Al Maqbali et al. revealed that approximately one-third of nurses working during the pandemic experienced psychological symptoms [4]. HCWs working in high-risk environments during emergencies, such as the COVID-19 outbreak, are more susceptible to mental health issues like stress, anxiety, and depression [5]. Research conducted among nurses in Hubei province showed that working in isolation wards was a risk factor for depression, anxiety, and stress [6]. Similar studies during previous outbreaks, such as SARS, also found that nurses experienced moderate psychological distress [7]. Additionally, COVID-19 has been identified as an independent risk factor for stress among HCWs [8].

Stress is an inevitable part of life, involving both psychological and physiological components. Chronic stress has been linked to aging and cancer development [9]. The implementation of mass quarantines during the COVID-19 pandemic has caused fear and anxiety among HCWs [10]. Nurses, in particular, have faced heightened stress due to the highly contagious nature of COVID-19. They worry about contracting the virus themselves and transmitting it to others [11]. Frontline health professionals, exposed to COVID-19, are at a higher risk of developing mental health issues [12]. Studies have shown that a significant proportion of HCWs have experienced mood and sleep disturbances during the COVID-19 outbreak [13]. Past research on individuals exposed to SARS outbreaks indicated that they were more likely to experience post-traumatic stress symptoms [14]. In disaster situations, such as the COVID-19 pandemic, nurses tend to face higher levels of adverse mental health outcomes compared to physicians [15].

Despite the risks of infection, nurses' commitment to their profession motivates them to continue caring for COVID-19 patients [16]. Overall, the COVID-19 pandemic has placed immense psychological pressure on HCWs, particularly nurses, due to the challenges they face on the frontline [17]. Understanding and addressing these stress-related issues are crucial to support the well-being and resilience of HCWs during this unprecedented global health crisis [18].

## Materials and methods

Study design

A cross-sectional survey-based descriptive design was conducted at a tertiary care teaching hospital (King Abdulaziz University Hospital, Jeddah) in the Kingdom of Saudi Arabia, designated to treat patients with COVID-19.

Sample

The participants in the study were nurses working in different units. We used convenience sampling to select study respondents. The sample consisted of 1044 nurses who were working in different nursing units such as General Wards, Critical Care Areas, Outpatient Departments, Operation Rooms, and Emergency Departments. The inclusion criteria were all nursing staff working currently in our setting and surveys with complete information and the exclusion criteria were surveys with incomplete information. Out of 1433 nurses, 1044 nurses responded to the survey and 4 were excluded due to incomplete information.

Ethical consideration

Ethical approval was obtained from the KAU Biomedical Research Ethics Committee. The participants were asked to answer a ‘YES’ or ‘NO’ question to confirm their willingness to participate voluntarily. After the confirmation, participants were directed to complete the self-reported questionnaire. Nurses were assured that their participation and the information provided would be anonymous and kept confidential.

Data collection

An online survey was conducted using Google Forms from July 18, 2021, to September 6, 2021. The questionnaire collected demographic data of the participants. The online survey link was sent to all nursing staff through the head nurses via email with the approval of the Nursing Director. Participants were informed about the study objectives. The data collection was divided into three sections: first, the perceived stress level of nurses during the COVID-19 outbreak, second the association of selected socio-demographic characteristics with perceived stress level and finally the identification of the three major causes of stress among nurses at KAUH during the COVID-19 pandemic. The Perceived Stress Scale (PSS) was used to assess nurses' stress levels. This scale is a widely used psychological instrument for measuring the perception of stress. It assesses the degree to which situations in one's life are appraised as stressful. Respondents rate items on a five-point Likert scale, ranging from never (0) to very often (4), with a total score range of 0 to 40. PSS scores are categorized as follows: 0 to 13 for low stress, 14 to 26 for moderate stress, and 27 to 40 for high perceived stress [19].

Data analysis

Data analysis was conducted using IBM SPSS Statistics for Windows, Version 21 (Released 2012; IBM Corp., Armonk, New York, United States). Descriptive analysis was used to calculate the mean and percentage. The association between socio-demographic characteristics and perceived stress level was assessed using the chi-square test and Fisher's exact test at 5% and 1% levels of significance. Univariate ordinal regression analysis was performed to assess the association of perceived stress levels with socio-demographic factors. Multivariable logistic regression was also employed to control for the socio-demographic factors of the participants. The study reported both unadjusted/crude odds ratio (COR) obtained from univariate analysis and adjusted odds ratio (AOR) obtained from multivariable logistic regression, which takes into account other covariates, including confounders.

## Results

Participant characteristics

A total of 1044 nursing professionals participated in the survey, with the majority being female (88.6%) and male respondents comprising only 11.4%. The distribution of participants across different age groups was as follows: below 25 years (1.1%), 25-30 years (21.4%), 31-35 years (31.8%), 36-40 years (19.5%), 41-45 years (10.7%), 46-50 years (8.5%), and above 50 years (7.9%); 76.6% of the participants were married, while 22.3% were single. 1.1% of participants held a master's degree, 529 (50.7%) had a bachelor's degree, and 504 (48.3%) had a diploma in nursing. The majority of the participants were from India (69.7%), followed by the Philippines (25.8%) and Saudi Arabia (3.2%). Seventy-seven percent of the staff were on shift duties, while 23.0% worked on straight shifts. Three hundred and sixteen participants (30.3%) had 5-10 years of experience, 27.6% had 11-15 years, 12.0% had 16-20 years, 22.2% had less than five years, and 8% had more than 20 years of experience. Out of the total participants, 79.0% had experience in dealing with COVID-19 patients. The study participants were categorized based on their roles as follows: Frontline Staff (83.6%), Middle Management (12.9%), Education and Training (2.2%), and Upper Management (1.2%).

Frequency and percentage distribution of perceived stress levels among nurses working during COVID-19

**Figure 1:**
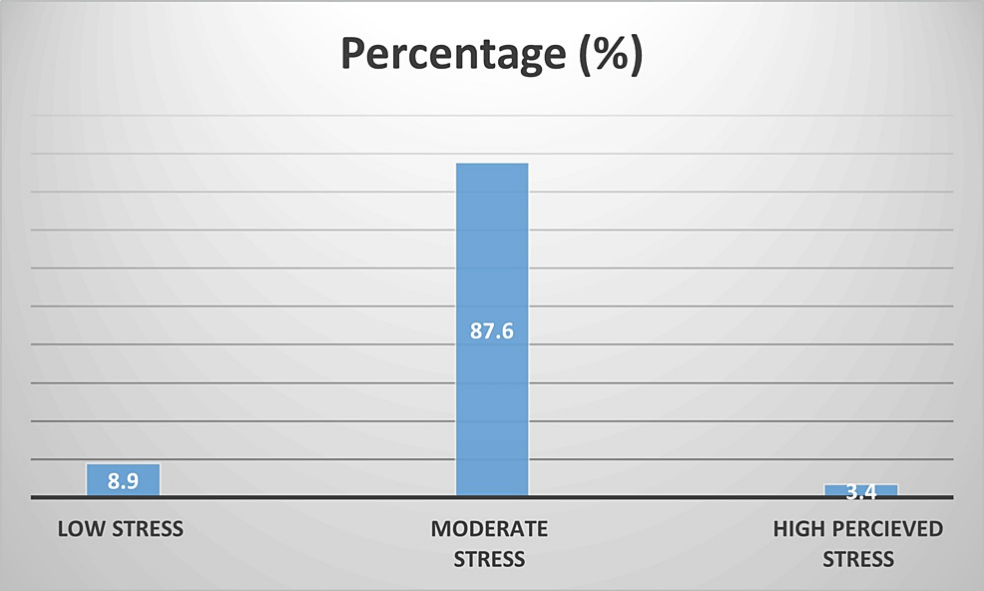
Frequency and Percentage Distribution of Level of Perceived Stress Among Nurses Working During COVID-19 COVID-19: Coronavirus disease 2019

Figure 1 displays the distribution of perceived stress levels among 1044 nursing professionals at KAUH during the COVID-19 pandemic. Of the participants, 93 (8.9%) experienced low levels of stress, 915 (87.6%) had a moderate level of stress, and 36 (3.4%) reported high perceived stress.

Frequency and percentage distribution of causes of stress

The study investigated 10 common causes of stress among nurses, and the results revealed that the three primary causes were as follows: workload: 284 (27.8%) of the participants reported workload as a significant cause of stress; lack of resources: 213 (20.9%) of the participants cited the lack of resources as a major stress factor; and fear of getting COVID-19: 126 (12.3%) of the participants expressed fear of contracting COVID-19 as a significant source of stress. These findings indicate that workload, lack of resources, and fear of COVID-19 are the top three stressors among nurses during the COVID-19 pandemic as shown in Table 1.

**Table 1:** Frequency and Percentage Distribution for Causes of Stress Among Nurses Working During COVID-19 (N=1044) COVID-19: Coronavirus disease 2019

What causes you the most stress on your job?	N(%)
Death and dying	32 (03.1)
Documentation	34 (03.3)
Fear of getting COVID-19	126 (12.3)
Follow up with other health care team to complete the required task	76 (07.4)
High demand from patient / family	70 (06.9)
High patient acuity	15 (01.5)
Lack of resources	213 (20.9)
Medication dispensing process	42 (04.1)
Pressure from immediate superiors	106 (10.4)
Workload	284 (27.8)
Others	23 (02.3)

Distribution of stress levels across socio-demographic characteristics 

It is evident that among the 1044 nursing professionals, there is a significant association between the level of stress and nationality (p-value= 0.006), work experience (p-value= 0.001), and experience with COVID patients (p-value= 0.05) as shown in Table 2. On the other hand, the following socio-demographic variables showed no significant association with stress levels: age, gender, marital status, educational level, unit, work schedule, and work status. These variables did not have a significant impact on the perceived stress levels of the nursing professionals in the study.

**Table 2:** Distribution of Stress Levels Across Socio-Demographic Characteristics *Significant at 0.05 level

Variable	Category	Stress Levels			Total	χ^2 ^value	p-value
		Low Stress	Moderate Stress	High Perceived Stress			
Age	25-30 Years	14	202	7	223	8.761	0.687
		15.1%	22.1%	19.4%	21.4%		
	31-35 Years	27	292	13	332		
		29.0%	31.9%	36.1%	31.8%		
	36-40 Years	22	172	10	204		
		23.7%	18.8%	27.8%	19.5%		
	41-45 Years	8	92	3	103		
		8.6%	10.1%	8.3%	9.9%		
	46-50 Years	10	77	2	89		
		10.8%	8.4%	5.6%	8.5%		
	Less than 25 Years	1	10	0	11		
		1.1%	1.1%	0.0%	1.1%		
	More than 50 Years	11	70	1	82		
		11.8%	7.7%	2.8%	7.9%		
Gender	Female	77	815	33	925	3.399	0.181
		82.8%	89.1%	91.7%	88.6%		
	Male	16	100	3	119		
		17.2%	10.9%	8.3%	11.4%		
Marital Status	Married	72	702	26	800	0.862	0.876
		77.4%	76.7%	72.2%	76.6%		
	Single	20	203	10	233		
		21.5%	22.2%	27.8%	22.3%		
	Others	1	10	0	11		
		1.1%	1.1%	0.0%	1.1%		
Educational Level	Bachelor's Degree	46	462	21	529	7.020	0.109
		49.5%	50.5%	58.3%	50.7%		
	Diploma	46	445	13	504		
		49.5%	48.6%	36.1%	48.3%		
	Master's Degree	1	8	2	11		
		1.1%	0.9%	5.6%	1.1%		
Nationality	India	64	645	19	728	16.781	0.006*
		68.8%	70.5%	52.8%	69.7%		
	Philippines	20	235	14	269		
		21.5%	25.7%	38.9%	25.8%		
	Saudi	5	27	1	33		
		5.4%	3.0%	2.8%	3.2%		
	Others	4	8	2	14		
		4.3%	0.9%	5.6%	1.3%		
Unit	Cath Lab	1	8	0	9	59.234	0.308
		1.1%	0.9%	0.0%	0.9%		
	CCU	4	31	1	36		
		4.3%	3.4%	2.8%	3.4%		
	Day Care	2	11	1	14		
		2.2%	1.2%	2.8%	1.3%		
	Dialysis	3	30	1	34		
		3.2%	3.3%	2.8%	3.3%		
	Endoscopy	1	18	0	19		
		1.1%	2.0%	0.0%	1.8%		
	ER	11	94	4	109		
		11.8%	10.3%	11.1%	10.4%		
	FMW	3	24	3	30		
		3.2%	2.6%	8.3%	2.9%		
	FSW	3	18	0	21		
		3.2%	2.0%	0.0%	2.0%		
	Hema/Onco	1	9	1	11		
		1.1%	1.0%	2.8%	1.1%		
	Isolation 1	2	17	1	20		
		2.2%	1.9%	2.8%	1.9%		
	Isolation 2	2	43	2	47		
		2.2%	4.7%	5.6%	4.5%		
	LD	3	21	0	24		
		3.2%	2.3%	0.0%	2.3%		
	MICU	8	89	4	101		
		8.6%	9.7%	11.1%	9.7%		
	MMW	2	40	1	43		
		2.2%	4.4%	2.8%	4.1%		
	MSW	0	14	1	15		
		0.0%	1.5%	2.8%	1.4%		
	NICS	0	8	0	8		
		0.0%	0.9%	0.0%	0.8%		
	NICU	8	50	3	61		
		8.6%	5.5%	8.3%	5.8%		
	NICU 2	1	6	0	7		
		1.1%	0.7%	0.0%	0.7%		
	Nursery	7	34	0	41		
		7.5%	3.7%	0.0%	3.9%		
	OB Gyane	0	16	0	16		
		0.0%	1.7%	0.0%	1.5%		
	OPD	4	41	1	46		
		4.3%	4.5%	2.8%	4.4%		
	OR	4	65	2	71		
		4.3%	7.1%	5.6%	6.8%		
	PCICU	5	42	2	49		
		5.4%	4.6%	5.6%	4.7%		
	Pedia 1	0	7	0	7		
		0.0%	0.8%	0.0%	0.7%		
	Pedia 2	4	43	0	47		
		4.3%	4.7%	0.0%	4.5%		
	PICU	1	48	1	50		
		1.1%	5.2%	2.8%	4.8%		
	PICU 2	4	3	0	7		
		4.3%	0.3%	0.0%	0.7%		
	Private	2	24	1	27		
		2.2%	2.6%	2.8%	2.6%		
	Radiotherapy	2	2	0	4		
		2.2%	0.2%	0.0%	0.4%		
	SICU	1	37	4	42		
		1.1%	4.0%	11.1%	4.0%		
	SIMCU	0	1	0	1		
		0.0%	0.1%	0.0%	0.1%		
	Others	4	21	2	27		
		4.3%	2.3%	5.6%	2.6%		
Work Schedule	Shift Duty	71	703	30	804	0.853	0.653
		76.3%	76.8%	83.3%	77.0%		
	Straight Duty	22	212	6	240		
		23.7%	23.2%	16.7%	23.0%		
Work Experience	5-10 Years	24	274	18	316	24.503	0.001*
		25.8%	29.9%	50.0%	30.3%		
	11-15 Years	23	257	8	288		
		24.7%	28.1%	22.2%	27.6%		
	16-20 Years	24	96	5	125		
		25.8%	10.5%	13.9%	12.0%		
	Less than 5 Years	16	211	5	232		
		17.2%	23.1%	13.9%	22.2%		
	More than 20 Years	6	77	0	83		
		6.5%	8.4%	0.0%	8.0%		
Experience with COVID patients - Taken care of covid -19 patients	No	28	186	5	219	6.001	0.05*
		30.1%	20.3%	13.9%	21.0%		
	Yes	65	729	31	825		
		69.9%	79.7%	86.1%	79.0%		
Work Status	Education and Training	2	21	0	23	1.670	0.912
		2.2%	2.3%	0.0%	2.2%		
	Frontline Staff	78	764	31	873		
		83.9%	83.5%	86.1%	83.6%		
	Middle Management	12	119	4	135		
		12.9%	13.0%	11.1%	12.9%		
	Upper Management	1	11	1	13		
		1.1%	1.2%	2.8%	1.2%		

Univariate ordinal logistic regression for socio-demographic factors affecting the stress level

Univariate ordinal logistic regression was used to assess the association of the perceived stress level with socio-demographic factors (Table 3). Among these factors, experience with COVID patients showed a significant association with the perceived stress level (COR = 5.86, 95% CI 0.380-9.03). This indicates that nursing professionals who have taken care of COVID-19 patients were 5.86 times more likely to report higher levels of perceived stress compared to those who have not taken care of COVID-19 patients, based on the unadjusted odds ratio. On the other hand, the following socio-demographic variables showed no significant association with stress levels: age, gender, marital status, educational level, unit, work schedule, and work status. These variables did not have a significant impact on the perceived stress levels of the nursing professionals in the study as shown in Table 3.

**Table 3:** Univariate Ordinal Logistic Regression for Factors Affecting the Stress Level OR: Odds ratio

Variables	Category	Estimate	Sig.	Unadjusted OR	95% Confidence Interval	
					Lower Bound	Upper Bound
Years	Less than 25	-0.397	0.654	0.672	0.118	3.815
	25-30	0.104	0.702	1.109	0.652	1.887
	36-40	-0.158	0.563	0.854	0.501	1.457
	41-45	-0.054	0.876	0.947	0.479	1.874
	46-50	-0.405	0.247	0.667	0.335	1.325
	More than 50	-0.641	0.065	0.527	0.267	1.040
	31-35	0^a^				
Gender	Male	-0.507	0.065	0.602	0.351	1.032
	Female	0^a^				
Marital Status	Single	0.126	0.580	1.135	0.725	1.777
	Others	-0.257	0.768	0.773	0.140	4.269
	Married	0^a^				
Educational Level	Diploma	-0.155	0.413	0.856	0.591	1.241
	Master's Degree	1.308	0.133	3.700	0.672	20.359
	Bachelor's Degree	0^a^				
Nationality	Philippines	0.356	0.114	1.427	0.918	2.219
	Saudi	-0.480	0.323	0.619	0.239	1.602
	Others	-0.754	0.270	0.470	0.123	1.798
	India	0^a^				
Unit	Cath Lab	-0.373	0.706	0.689	0.099	4.781
	CCU	-0.167	0.773	0.846	0.272	2.629
	Day Care	-0.091	0.916	0.913	0.168	4.954
	Dialysis	0.056	0.928	1.057	0.318	3.516
	Endoscopy	0.130	0.868	1.139	0.244	5.318
	FMW	0.652	0.337	1.920	0.507	7.272
	FSW	-0.603	0.355	0.547	0.152	1.963
	Hema/Onco	0.652	0.534	1.920	0.245	15.019
	Isolation 1	0.130	0.866	1.139	0.252	5.144
	Isolation 2	0.652	0.255	1.920	0.625	5.896
	LD	-0.477	0.453	0.621	0.179	2.156
	MICU	0.240	0.584	1.271	0.539	2.997
	MMW	0.412	0.481	1.511	0.480	4.752
	MSW	1.304	0.129	3.686	0.683	19.898
	NICS	0.652	0.592	1.920	0.177	20.841
	NICU	-0.171	0.723	0.842	0.327	2.171
	NICU 2	-0.603	0.565	0.547	0.070	4.263
	Nursery	-0.786	0.113	0.456	0.173	1.203
	OB Gyane	0.652	0.462	1.920	0.338	10.910
	OPD	0.000	1.000	1.000	0.344	2.905
	OR	0.361	0.462	1.434	0.549	3.749
	PCICU	0.026	0.961	1.026	0.360	2.924
	Pedia 1	0.652	0.615	1.920	0.151	24.330
	Pedia 2	-0.162	0.758	0.850	0.303	2.387
	PICU	0.652	0.244	1.920	0.641	5.753
	PICU 2	-2.587	0.002	0.075	0.015	0.376
	Private	0.267	0.699	1.306	0.337	5.063
	Radiotherapy	-2.301	0.027	0.100	0.013	0.773
	SICU	1.363	0.017	3.907	1.278	11.943
	Others	-0.118	0.856	0.888	0.248	3.186
	ER	0^a^				
Work Schedule	Straight Duty	-0.131	0.553	0.877	0.569	1.352
	Shift Duty	0^a^				
	Less than 5 Years	-0.262	0.338	0.769	0.450	1.316
Work Experience	11-15 Years	-0.308	0.232	0.735	0.443	1.218
	16-20 Years	-1.107	0.000	0.331	0.184	0.595
	More than 20 Years	-0.471	0.212	0.624	0.298	1.308
	5-10 Years	0^a^				
Experience with COVID patients	No	-0.534	0.015	0.586	0.380	0.903
	Yes	0^a^				
Work Status	Education and Training	-0.257	0.674	0.773	0.233	2.563
	Middle Management	-0.045	0.872	0.956	0.553	1.653
	Upper Management	0.498	0.574	1.646	0.289	9.365
	Frontline Staff	0^a^				
Variables	Category	Estimate	Sig.	Unadjusted OR	95% Confidence Interval	
					Lower Bound	Upper Bound
Years	Less than 25	-0.397	0.654	0.672	0.118	3.815
	25-30	0.104	0.702	1.109	0.652	1.887
	36-40	-0.158	0.563	0.854	0.501	1.457
	41-45	-0.054	0.876	0.947	0.479	1.874
	46-50	-0.405	0.247	0.667	0.335	1.325
	More than 50	-0.641	0.065	0.527	0.267	1.040
	31-35	0^a^				
Gender	Male	-0.507	0.065	0.602	0.351	1.032
	Female	0^a^				
Marital Status	Single	0.126	0.580	1.135	0.725	1.777
	Others	-0.257	0.768	0.773	0.140	4.269
	Married	0^a^				
Educational Level	Diploma	-0.155	0.413	0.856	0.591	1.241
	Master's Degree	1.308	0.133	3.700	0.672	20.359
	Bachelor's Degree	0^a^				
Nationality	Philippines	0.356	0.114	1.427	0.918	2.219
	Saudi	-0.480	0.323	0.619	0.239	1.602
	Others	-0.754	0.270	0.470	0.123	1.798
	India	0^a^				
Unit	Cath Lab	-0.373	0.706	0.689	0.099	4.781
	CCU	-0.167	0.773	0.846	0.272	2.629
	Day Care	-0.091	0.916	0.913	0.168	4.954
	Dialysis	0.056	0.928	1.057	0.318	3.516
	Endoscopy	0.130	0.868	1.139	0.244	5.318
	FMW	0.652	0.337	1.920	0.507	7.272
	FSW	-0.603	0.355	0.547	0.152	1.963
	Hema/Onco	0.652	0.534	1.920	0.245	15.019
	Isolation 1	0.130	0.866	1.139	0.252	5.144
	Isolation 2	0.652	0.255	1.920	0.625	5.896
	LD	-0.477	0.453	0.621	0.179	2.156
	MICU	0.240	0.584	1.271	0.539	2.997
	MMW	0.412	0.481	1.511	0.480	4.752
	MSW	1.304	0.129	3.686	0.683	19.898
	NICS	0.652	0.592	1.920	0.177	20.841
	NICU	-0.171	0.723	0.842	0.327	2.171
	NICU 2	-0.603	0.565	0.547	0.070	4.263
	Nursery	-0.786	0.113	0.456	0.173	1.203
	OB Gyane	0.652	0.462	1.920	0.338	10.910
	OPD	0.000	1.000	1.000	0.344	2.905
	OR	0.361	0.462	1.434	0.549	3.749
	PCICU	0.026	0.961	1.026	0.360	2.924
	Pedia 1	0.652	0.615	1.920	0.151	24.330
	Pedia 2	-0.162	0.758	0.850	0.303	2.387
	PICU	0.652	0.244	1.920	0.641	5.753
	PICU 2	-2.587	0.002	0.075	0.015	0.376
	Private	0.267	0.699	1.306	0.337	5.063
	Radiotherapy	-2.301	0.027	0.100	0.013	0.773
	SICU	1.363	0.017	3.907	1.278	11.943
	Others	-0.118	0.856	0.888	0.248	3.186
	ER	0^a^				
Work Schedule	Straight Duty	-0.131	0.553	0.877	0.569	1.352
	Shift Duty	0^a^				
	Less than 5 Years	-0.262	0.338	0.769	0.450	1.316
Work Experience	11-15 Years	-0.308	0.232	0.735	0.443	1.218
	16-20 Years	-1.107	0.000	0.331	0.184	0.595
	More than 20 Years	-0.471	0.212	0.624	0.298	1.308
	5-10 Years	0^a^				
Experience with COVID patients	No	-0.534	0.015	0.586	0.380	0.903
	Yes	0^a^				
Work Status	Education and Training	-0.257	0.674	0.773	0.233	2.563
	Middle Management	-0.045	0.872	0.956	0.553	1.653
	Upper Management	0.498	0.574	1.646	0.289	9.365
	Frontline Staff	0^a^				

Multivariate ordinal logistic regression for socio-demographic factors affecting the stress level

The multivariable ordinal logistic regression analysis (Table 4) was conducted to assess the combined effect of socio-demographic factors on stress levels. The likelihood ratio chi-square test resulted in a significant p-value of 0.024, indicating that the model as a whole (gender and nationality as predictors) was statistically significant. This suggests that the combination of gender and nationality had a significant effect on stress levels among nursing professionals. Regarding individual predictors, the analysis found that both gender and nationality had significant effects on stress levels after controlling for other factors. first gender, the AOR for gender was 0.319, with a 95% confidence interval (CI) of 0.166-0.613. This indicates that, after adjusting for other factors, female nursing professionals were 68.1% less likely to report higher levels of perceived stress compared to male nursing professionals. Second is the nationality; the analysis showed that the stress levels of nurses from the Philippines were significantly different from those of Indian nurses. The AOR for Filipino nurses was 2.559, with a 95% CI of 1.297-5.051. This means that, after adjusting for other factors, Filipino nursing professionals were 2.559 times more likely to report higher levels of perceived stress compared to Indian nursing professionals. These results indicate that both gender and nationality are important factors that influence the perceived stress levels among nursing professionals. Female nurses were less likely to report high stress levels compared to male nurses, and there was a significant difference in stress levels between nurses from the Philippines and Indian nurses. It is essential to consider these findings while designing interventions and support systems to address stress and improve the well-being of nursing professionals, especially during challenging times like the COVID-19 pandemic.

**Table 4:** Multivariable Ordinal Logistic Regression for Socio-Demographic Factors Affecting the Stress Level 0^a^ - reference; OR: Odds ratio

Variables	Category	B	p-value		Adjusted OR	95% Wald Confidence Interval	
			Category	Overall		Lower	Upper
Age	Less than 25 Years	-0.452	0.644	0.920	0.636	0.093	4.339
	25-30 Years	0.133	0.692		1.142	0.591	2.207
	36-40 Years	0.211	0.510		1.235	0.659	2.315
	41-45	0.294	0.510		1.342	0.559	3.224
	46-50 Years	-0.028	0.953		0.972	0.379	2.491
	More than 50 Years	-0.246	0.664		0.782	0.258	2.369
	31-35 Years	0^a^			1		
Gender	Male	-1.142	0.001	0.001	0.319	0.166	0.613
	Female	0^a^			1		
Marital Status	Single	-0.135	0.643	0.873	0.873	0.492	1.549
	Others	-0.267	0.778		0.766	0.119	4.921
	Married	0^a^			1		
Educational Level	Diploma	0.114	0.690	0.178	1.121	0.640	1.963
	Master's Degree	1.855	0.055		6.389	0.965	42.314
	Bachelor's Degree	0^a^			1		
Nationality	Philippines	0.940	0.007	0.016	2.559	1.297	5.051
	Saudi	-0.646	0.268		0.524	0.167	1.644
	Others	0.018	0.984		1.018	0.174	5.957
	India	0^a^			1		
Unit	Cath Lab	-0.457	0.670	0.296	0.633	0.077	5.192
	CCU	-0.100	0.866		0.905	0.284	2.889
	Day Care	-0.283	0.763		0.754	0.120	4.735
	Dialysis	-0.073	0.913		0.929	0.252	3.433
	Endoscopy	-0.201	0.810		0.818	0.158	4.230
	FMW	0.477	0.509		1.611	0.391	6.641
	FSW	-0.674	0.322		0.510	0.134	1.937
	Hema/Onco	0.505	0.658		1.657	0.177	15.520
	Isolation 1	-0.175	0.827		0.839	0.174	4.040
	Isolation 2	0.331	0.576		1.392	0.436	4.442
	LD	-0.542	0.422		0.582	0.155	2.184
	MICU	0.300	0.502		1.350	0.562	3.240
	MMW	0.635	0.281		1.888	0.594	5.996
	MSW	1.350	0.117		3.857	0.714	20.842
	NICS	0.551	0.653		1.735	0.157	19.212
	NICU	-0.222	0.685		0.801	0.274	2.343
	NICU 2	-0.422	0.702		0.656	0.075	5.700
	Nursery	-1.017	0.064		0.362	0.123	1.061
	OB Gyane	0.326	0.706		1.385	0.255	7.519
	OPD	-0.192	0.751		0.825	0.252	2.705
	OR	0.438	0.377		1.550	0.586	4.101
	PCICU	-0.019	0.975		0.981	0.307	3.142
	Pedia 1	0.823	0.523		2.278	0.182	28.520
	Pedia 2	-0.073	0.897		0.930	0.310	2.788
	PICU	0.555	0.346		1.741	0.549	5.525
	PICU 2	-2.401	0.005		0.091	0.017	0.492
	Private	0.015	0.983		1.015	0.256	4.031
	Radiotherapy	-2.808	0.017		0.060	0.006	0.611
	SICU	1.403	0.017		4.067	1.291	12.811
	SIMCU	0.009	0.998		1.009	0.001	1000.638
	Others	-0.334	0.637		0.716	0.179	2.862
	ER	0^a^			1		
Work Schedule	Straight Duty	0.271	0.388	0.386	1.311	0.709	2.423
	Shift Duty	0^a^			1		
	Less than 5 Years	-0.129	0.672	0.072	0.879	0.483	1.599
Work Experience	11-15 Years	-0.495	0.115		0.610	0.329	1.128
	16-20 Years	-1.250	0.005		0.286	0.120	0.682
	More than 20 Years	-0.586	0.308		0.557	0.181	1.716
	5-10 Years	0^a^			1		
Experience with COVID patients	No	-0.391	0.168	0.169	0.676	0.388	1.179
	Yes	0a			1		
Work Status	Education and Training	-0.440	0.500	0.898	0.644	0.179	2.314
	Middle Management	0.002	0.996		1.002	0.545	1.840
	Upper Management	0.372	0.709		1.451	0.205	10.247
	Frontline Staff	0^a^			1		

## Discussion

Perceived stress level of nurses during the COVID-19 outbreak

The findings of this study reveal that among 1044 nursing professionals in our setting, 87.6% had a moderate level of stress, and 3.4% experienced high perceived stress during the COVID-19 pandemic. The average stress score obtained was 18.8 ± 4.1, indicating a moderate level of self-perceived stress. These results are consistent with similar studies conducted in different regions during the pandemic. Studies from South Korea, Wuhan (China), and Medina (Saudi Arabia) reported stress scores around the same range [20-22]. Other studies have also highlighted the high levels of stress, anxiety, and depression among frontline HCWs caring for COVID-19 patients [23]. The elevated stress levels observed among nurses can be attributed to factors such as increased workload, fear of getting infected, and the emotional toll of caring for critically ill patients during a pandemic [24].

Association of selected socio-demographic characteristics and perceived stress levels

The study identified significant associations between perceived stress levels and certain socio-demographic variables such as nationality, work experience, and experience with COVID-19 patients. However, no significant associations were found with age, gender, marital status, education, unit, work schedule, and work status.

Gender

The analysis revealed significant gender differences in stress levels during the COVID-19 pandemic, with female nursing professionals reporting lower stress levels compared to males. This finding is consistent with several other studies that have reported higher stress levels among male HCWs compared to females [25,26]. However, it is worth noting that there have been conflicting results in some studies, with others reporting higher stress levels among females [27]. The reasons behind these gender differences in stress perception may be multifactorial and warrant further investigation.

Nationality

Nationality was another significant predictor of stress levels among nursing professionals in the study. This study describes that nurses from the Philippines were more stressed than others. In the current study, a high perceived stress level was observed more in Philippine nurses than in Indian and Saudi nurses. This difference could be attributed to variations in cultural norms, coping mechanisms, and support systems, which can influence how individuals perceive and manage stress during challenging situations. Similar to our study another study from Saudi Arabia also found that Philippine nurses had more negative feelings and experienced more factors causing stress compared to other nationalities [28]. In contrast to the current study, the other two studies from India [29] and Saudi Arabia [30] showed that Indian nurses had higher stress levels compared with nurses from other nationalities. It may be due to the family-oriented structure of Indian society [31], which might increase their homesickness due to the environmental changes. Another study by Bhatia et al., among 87 nurses working in two of the tertiary care teaching hospitals of Central Delhi, found that the majority (87.4%) of nurses reported their job as stressful [32]. Similar to the current study, a nationwide observational study conducted among 433 healthcare practitioners in India shows that the prevalence of high-level stress was low, only 3.7% (45), and another multicenter study from the Asia-Pacific region, including HCWs from major tertiary healthcare institutions from India, Singapore, Malaysia, Vietnam, and Indonesia, India showed the lowermost prevalence of adverse psychological effects [33]. On the other hand, another study from Saudi Arabia shows no significant association between nurses' stress levels and nationality [34].

Work experience

The current study shows a high level of stress with moderately experienced nurses. The high perceived stress level was observed more in the nurses with an experience of 5-10 years (50.0%) than those experienced 11-15 years (22.2%), 16-20 years, and less than five years of experience (13.9%). Nurses with more than 20 years of experience have not been exposed to high levels of stress. The association between the perceived stress level and work experience (p = 0.001) remained significant in the analysis. Similarly, a study among HCWs from China revealed an increased risk of developing psychological stress among HCWs with 10 years or more of work experience [35]. In contrast to the current study, a study conducted in Turkey shows that those who were younger and had fewer years of work experienced significantly more stress than their counterparts who had worked for longer [36]. Similarly, a study among oncology nurses shows that the demographic variables of age and work experience in nursing showed a substantial positive association with work-related stress scores [37]. Also, a study from Pakistan reported job experience as an important forecaster, where nurses with less experience were more likely to develop depression, anxiety, and stress [38]. Another study by Humpel & Caputi also concluded that nurses with shorter experience displayed more depression, anxiety, and stress [39]. Another study from Iran also indicated that nurses with fewer years of work experience show more stress while more experienced nurses exhibit more resilience [40]. Therefore, it needs further research to find out the reason behind high levels of stress among moderately experienced nurses, which is different from other studies around the world. The hypothesis for this variation might be because the majority of our study participants were females (88.6%) and between the ages of 25-35 years (53%), which is supported by several studies that females and young age groups are prone to mental stress compared to male gender and older adults [41-45]. In addition, this may be because this group of nurses in our setting were assigned as senior nurses need to make more decisions regarding the care of COVID-19 patients and therefore possessed more responsibilities and pressure.

Experience with COVID-19 patients

High perceived stress levels were observed more in professionals who have taken care of COVID-19 patients (86.1%) than those who haven’t (13.9%). A moderate level of stress was also observed more in professionals who have taken care of COVID-19 patients (79.7%) than those who haven’t (20.3%). The association between the perceived stress level and experience with COVID-19 patients (p = 0.05) remained significant in the analysis. Experience with COVID patients or taking care of COVID-19 patients (COR = 5.86 (95% CI 0.380-9.03) was found to be a significant factor in univariate analysis. Similarly, a study among 218 HCWs from Italy found that HCWs assisting patients with COVID-19 acquired significantly much higher scores on depression, anxiety, and stress than other participants [46]. A study involving a sample of 1257 Chinese HCWs reported that the frontline HCWs involved in direct diagnosis, treatment, and care of patients with COVID-19 were associated with a higher risk of symptoms of depression, anxiety, insomnia, and distress [47]. Another study from Italy among HCWs assisting patients with COVID-19 displayed work-related psychological pressure, emotional exhaustion, and somatic symptoms [48]. A study from Kenya among 255 nurses shows that depression, anxiety, insomnia, distress, and burnout were reported in 45.9%, 48.2%, 37.0%, 28.8%, and 47.9% of all nurses. Also, the study displays that nurses directly involved with COVID-19 patients reported higher rates of mental health symptoms [49]. A study with the participation of 125 nurses working in COVID-19 wards in Iran displays that the rate of depression, anxiety, and stress in nurses working in COVID-19 wards is moderate. [50]. Other studies also report that HCWs/nurses working with COVID-19 patients exhibit moderate rates of stress [51,52]. In contrary to the present study, a study from Turkey displays no significant relationship between psychological symptoms and the organization or working units or COVID-19 services [53]. 

Major causes of stress among nurses during the COVID-19 pandemic

In the present study, the major three causes of stress raised by the nurses were workload (27.8%), lack of resources (20.9%), and fear of getting COVID-19 (12.3%). Similar to this study a study by Cai et al. from China says that the COVID-19 epidemic in Hubei caused an increased workload and stress for medical staff and the factors associated with stress included the risk of infection to themselves and their families and increased patient mortality [54]. Studies from India, Palestine, Australia, and Iran even before the COVID-19 Pandemic, show that the major stressors experienced by nurses were death and dying patients and excessive workload [55-58]. A study from Malaysia also shows that workload and manpower availability are the major stressors among nurses [59]. A study conducted in central China showed death and dying, workload, and inadequate preparation as the most common sources of nursing stress [60]. Another study among oncology nurses also declares workload and patient death and dying are the main causes of stress [61]. The current study findings are in line with the study conducted by Purcell et al., which declares that patient workload and day of the week worked are important factors affecting nurses' stress levels [62]. A study conducted at the Department of Critical Care Medicine, Wuhan Pulmonary Hospital during the peak of the COVID-19 pandemic shows that the front-line nurses carried enormous workload, enduring fatigue, infection risk, and frustration with the death of patients as the factors causing high stress among them [63]. A comparative study conducted between a COVID-19 and non-COVID-19 hospital in Egypt displays Workload, dealing with death and dying, individual demands and worries, employing strict biosecurity measures, and disgrace represented the high-priority stressors among nurses of COVID hospitals [64]. Similar to this study, a study among nurses from a major university hospital in China, who responded to a national call to go to help the epic entre in early February 2020, included working in an isolated environment, PPE shortage, and the discomfort of pronged usage, sleep problems, the intensity of workload, cultural and language barriers, lack of family support, fear of being infected, and insufficient work experiences with COVID-19 as significant stressors [21]. A qualitative study from a center similar to our setting says that the nursing care of patients with COVID-19 was challenging and strenuous. The variety of patient care needs and an inadequate number of nursing staff increase the nurses' workload and physical fatigue [65]. Another supporting finding can be seen in a study from South Korea which mentions that the PSS-10 score was significantly connected with the fear of COVID-19 infection and fear of blame for COVID-19 infection [20]. A study by Chaudhari et al. also found that the Common causes of stress were conflicts with supervisors, unreasonable demands from patients and their families, workload, and dealing with death and dying [29]. Contrary to the present study, a study from Hubei province in China involving 1208 HCWs demonstrates that the main source of stress among frontline HCWs caring for COVID-19 patients is fear of being infected and the uneasiness caused by protective equipment [66].

## Conclusions

The first wave of COVID-19 caused significant psychological stress for nurses, including safety concerns, uncertainty about the disease, stigma, and social isolation. This study highlights the significant stress levels experienced by nursing professionals during the COVID-19 pandemic. The findings underscore the importance of addressing the mental health and well-being of nurses, as they are critical frontline HCWs facing unprecedented challenges. The association between stress levels and nationality, work experience, and experience with COVID-19 patients emphasizes the need for tailored interventions and support systems to meet the unique needs of different subgroups of nursing professionals. Healthcare institutions and policymakers should prioritize the implementation of evidence-based strategies to support nurses' mental health, such as providing access to mental health services, stress management training, and creating supportive work environments. Further research is warranted to explore the underlying factors contributing to the observed gender and nationality-based differences in stress levels among nursing professionals. By gaining a deeper understanding of these factors, targeted interventions can be developed to enhance the well-being and resilience of nurses, ultimately benefiting both healthcare providers and patients alike.
